# Exploring motor skill acquisition in bimanual coordination: insights from navigating a novel maze task

**DOI:** 10.1038/s41598-024-69200-1

**Published:** 2024-08-14

**Authors:** Miguel Cienfuegos, Jonathan Maycock, Abdeldjallil Naceri, Tobias Düsterhus, Risto Kõiva, Thomas Schack, Helge Ritter

**Affiliations:** 1 Neurocognition and Action - Biomechanics Group, Bielefeld University, 33615 Bielefeld, Germany; 2 Margin UG, 33602 Bielefeld, Germany; 3 Munich Institute of Robotics and Machine Intelligence (MIRMI), Technical University of Munich, 80992 Munich, Germany; 4 Neuroinformatics Group, Bielefeld University, 33619 Bielefeld, Germany

**Keywords:** Bimanual coordination, Motor learning, Kinematic dynamics, Tactile feedback, Maze, Motor control, Sensorimotor processing, Navigation

## Abstract

In this study, we introduce a novel maze task designed to investigate naturalistic motor learning in bimanual coordination. We developed and validated an extended set of movement primitives tailored to capture the full spectrum of scenarios encountered in a maze game. Over a 3-day training period, we evaluated participants’ performance using these primitives and a custom-developed software, enabling precise quantification of performance. Our methodology integrated the primitives with in-depth kinematic analyses and thorough thumb pressure assessments, charting the trajectory of participants’ progression from novice to proficient stages. Results demonstrated consistent improvement in maze performance and significant adaptive changes in joint behaviors and strategic recalibrations in thumb pressure distribution. These findings highlight the central nervous system’s adaptability in orchestrating sophisticated motor strategies and the crucial role of tactile feedback in precision tasks. The maze platform and setup emerge as a valuable foundation for future experiments, providing a tool for the exploration of motor learning and coordination dynamics. This research underscores the complexity of bimanual motor learning in naturalistic environments, enhancing our understanding of skill acquisition and task efficiency while emphasizing the necessity for further exploration and deeper investigation into these adaptive mechanisms.

## Introduction

In the realm of motor action, the phenomenon of bimanual manipulation holds paramount significance, necessitating the meticulous coordination of both hands. These manipulative endeavors can be delineated into two distinct paradigms: symmetrical manipulation, wherein both hands exhibit congruent contributions, exemplified by actions like coordinated object transportation or synchronous motion execution; and asymmetrical manipulation, typified by disparate contributions between hands. Examples of the latter include scenarios where one hand interfaces with a precision tool while the other operates a keyboard, or when one hand unevenly collaborates with its environment.

Despite the prevalence of bimanual tasks in our daily lives, there remains a substantial interest in understanding their underlying behavioral and learning mechanisms^1,2^. While a significant body of bimanual research has focused on tasks such as finger tapping or synchronized flexion and extension of fingers, which have advanced our comprehension of the neural regions governing bimanual coordination and their interplay, this body of work represents only part of the extensive research landscape^3–9^. Numerous studies over the past decades have explored various aspects of bimanual coordination, including movement frequency^10,11^, movement amplitude^4,12^, multifrequency coordination^13,14^, and two-hand reaching and grasping^5,15^, providing valuable insights into the neural and behavioral control of these activities.

Existing research on bimanual tasks requiring learning has emphasized rhythmic movements utilizing tools like joysticks^16,17^, or crank handles for screen drawing^18^. These studies have demonstrated condition-specific improvements in performance following thorough, repeated training. Despite these examples, there is still much to learn about the intricate nuances of cooperative bimanual interactions and their learning trajectories (refer to Obhi^1^ for a comprehensive review highlighting this gap). Recent studies have begun to explore more complex cooperative tasks, such as those where one hand supports the other^19,20^, or tasks involving navigation through detailed circuitry paths with a cursor^21^. The development of novel experimental setups^22–24^ indicates a growing interest in the domain of complex bimanual coordination.

Historically, motor learning studies that zero in on natural behaviors have been sparse, often leaving a gap in our understanding of adaptations in real-world settings instead of controlled lab environments^25^. In response, our study, aligning with the emerging trend of naturalistic experiments in motor learning and neuroscience^26^, aims to complement and extend this wave of experiments introducing a focus on bimanual tasks. We introduce a novel bimanual maze task that closely simulates real-world activities to deepen our understanding of how individuals learn and adapt to complex bimanual tasks. This endeavor not only aims to enrich the description of bimanual movements and their learning trajectories but also to shed light on the brain’s coordination of cooperative bimanual interactions and the management of multiple degrees of freedom (DOFs).

Bimanual tasks, such as the ones in our study, typically engage a wider network of brain regions compared to unimanual counterparts^27^, and they are especially relevant as they mirror complex real-world motor activities, offering profound insights into brain–behavior relationships^28^. Only a limited number of studies have investigated natural behaviors without significant simplification. For instance, Haar and colleagues explored whole-body coordination through pool playing to grasp the variability in muscular and biomechanical coordination^29^. Meanwhile, studies like that of Krotov and team dissected the art of striking a target with a whip in both discrete and rhythmic manners^30^. Other works have probed real-world tasks like dart throwing^31^ and juggling^32^ to discern the relationship between task-specific variables and performance parameters. Our study contributes to this evolving field by examining the intricate interactions and learning processes within a complex bimanual task, aiming to address the existing knowledge gap in naturalistic bimanual motor learning tasks.

**Figure 1:**
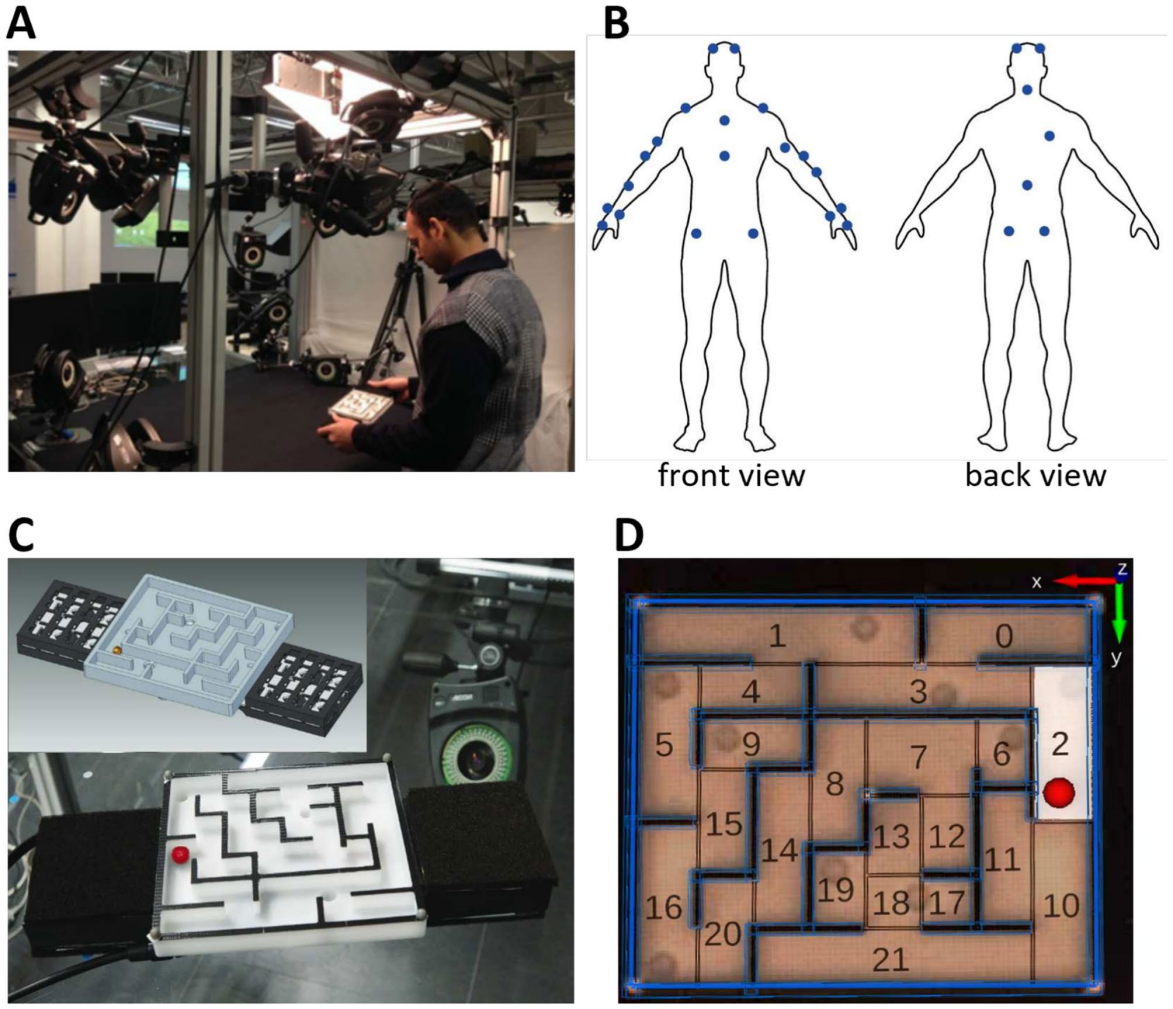
Experimental task and setup: (**A**) Twelve subjects participated in the study, performing a repeated task over three consecutive days for 20 min each. Their objective was to maneuver a red sphere through the maze from the starting position to the target position while ensuring there was no contact with the walls and avoiding falling into any pits. (**B**) The participants’ upper-body movement was tracked by attaching retro-reflective markers based on the Vicon Nexus plug-in-gait model from Vicon Motion Systems Ltd. (**C**) Novel tactile sensors were employed to monitor the finger pressure at the points where the hands made contact with the maze. (**D**) The movement of the metallic red sphere was tracked using a high-speed camera from Basler. Custom-made software was used to overlay a model of the maze, divided into 21 sections, onto the maze image, enabling accurate tracking of the sphere’s movement in each segment.

In our exploration of human manual skill acquisition, we are particularly interested in tasks that require a seamless orchestration of rapid sensorimotor actions. These actions rely on the synergy of tactile, kinesthetic, and visual sensing modalities. Drawing inspiration from the frameworks of Johansson and Flanagan^33,34^, we view manipulation tasks as sequences of sensory events intricately tied to specific subgoals, where the brain selects and deploys appropriate action-phase controllers^35,36^. To delve into these dynamics firsthand, we have developed a novel maze task where participants navigate a rolling sphere through a maze interspersed with obstacles. This task is bi-manually orchestrated (see Fig. 1A). Serving as a microcosm of real-world skills, this task demands intricate coordination of vision and haptic feedback from both hands^34^. This coordination is captured in detail thanks to the integration of advanced motion capture technology, which meticulously tracks both the maze and the sphere^37^, and the employment of pioneering tactile sensors^38^. This enhanced setup enables us to closely monitor the sphere motion and the finger forces exerted during the maze navigation^37,38^, offering a multidimensional view of the participants’ interaction with the maze.

In this paper, we introduce the maze platform in detail, elucidating the intricacies of its design and our approach to the automatic segmentation of trials. Central to our investigation is the comprehensive framework of measurements devised to evaluate participants’ performance within this complex environment. This evaluative framework is multi-dimensional, covering a spectrum of variables that capture the essence of motor skill acquisition. We have identified specific motor ‘primitives’^39^—the fundamental actions or sequences essential for navigating the maze—alongside kinematic movements that reveal the fluidity and precision of physical maneuvers. Tactile patterns are also scrutinized, providing insights into how participants adjust their grip and apply pressure through touch-sensitive interfaces.

Our study is guided by a series of hypotheses aimed at dissecting the mechanisms of skill acquisition in our novel maze task. We anticipate observing performance improvements as participants engage in repeated practice sessions^40^, with these advancements manifesting as increased proficiency and efficiency. To quantify these improvements and their impact on overall maze performance, we employ a primitives framework, which serves as an essential tool for our analysis.

Furthermore, our prediction is to observe a significant transformation in movement dynamics. To probe this phenomenon in depth, we scrutinize the velocity profiles of the joints and the correlation between joint movements. These metrics allow us to observe the adaptation in the dynamics of movement, a cornerstone of learning^41^. From the onset of participants’ interaction with the maze to the conclusion of the experiment, we anticipate witnessing substantial kinematic adaptations that are intricately linked to skill development, revealing skill acquisition in concrete terms.

Additionally, we hypothesize a decrease in average finger pressure exerted on the tactile sensors as participants gain expertise. This expected trend reflects the intricate interplay between stiffness and predictive control during the learning process^42^. Initially, participants may exhibit increased stiffness, manifested as higher fingertip pressure, to counterbalance errors encountered in navigating the maze. However, as participants’ proficiency improves and the frequency of errors diminishes, a corresponding decrease in stiffness is anticipated. We aim to document this phenomenon through a detailed analysis of thumb pressure distribution, capturing how adaptations in tactile feedback correlate with the progression of skill mastery.

Crucially, our analysis tracks the evolution of these measurements throughout the skill acquisition phase, capturing not just static snapshots of ability but the dynamic process of learning and adaptation. These metrics allow us to trace the trajectory of skill development offering a granular view of how complex bimanual coordination is mastered and sheds light on the processes by which the central nervous system coordinates a plethora of motor outputs in tandem with sensory feedback. Our research, rooted in these findings and centered around the maze paradigm, aims to expand the boundaries of current understanding in motor skill acquisition, integrating within the broader context of established motor behavior theories and upper limb coordination dynamics^43–45^.

## Results

Twelve volunteers, with little to no prior experience in similar maze games, participated in the study. Over the course of three consecutive days, they completed approximately 175 trials, with a 20-min learning session each day. The task involved guiding a sphere through the maze, with the objective of reaching the target position while avoiding pits and any contact with the walls (Fig. 1A). Throughout the learning process, we recorded the participants’ upper-body movements using the Vicon Nexus Plug-in-Gait model (Fig. 1B), collected tactile pressure data from sensors embedded in the maze (Fig.  1C), and tracked the sphere’s movement using a high-speed camera to evaluate performance on each trial (Fig.  1D).

### Cognitive primitives in maze task

**Figure 2:**
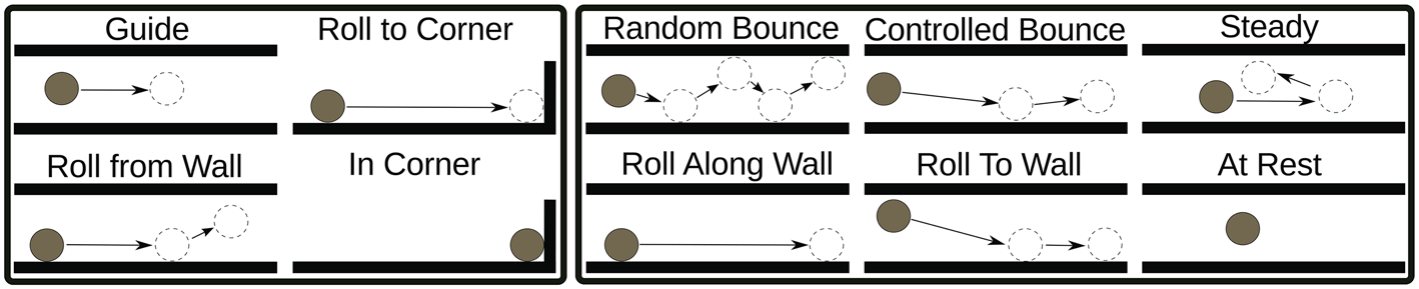
The cognitive primitives are divided into two sets. The left-hand side box presents the original primitives proposed by Bentivegna et al.^46,47^, while the right-hand box shows the extended set of primitives that we propose to encompass a wider range of movements and scenarios encountered during maze game performance.

Prior research has established a set of primitives derived from observing humans playing a maze game and engaging in self-practice. Bentivegna and colleagues posited that without employing primitives for learning, generalization is unattainable and that primitives enable the reuse of learned actions. The primitives they identified are: “Guide”, “Roll To Corner”, “Roll From Wall”, and “In Corner” (Fig. 2). These primitives represented sequences of task states, including the position and velocity of the sphere, as well as the maze tilt angles. However, these primitives were derived solely from successful trials and did not account for scenarios involving mistakes or variations in sensorimotor control.

To address this limitation and provide a more comprehensive representation of the maze game, we introduced the concept of “cognitive primitives”. Our definition of cognitive primitives refers to fundamental units of cognitive and motor actions that represent specific movement patterns and strategies in the context of the maze task. While other frameworks have explored cognitive primitives in the contexts of learning^48^ and language^49^, our approach aligns with the concept of action primitives as elementary building blocks for action representation, which is well-supported in the literature^50–55^.

We expanded the original set to include additional primitives: “Random Bounce”, “Controlled Bounce”, “Steady”, “Roll Along Wall”, “Roll To Wall”, and “At Rest” (Fig. 2). These new primitives encompass a wider range of movements, including bouncing, rolling along walls, resting, and controlled maneuvers. We aim to capture a broader spectrum of actions and accommodate scenarios involving mistakes or imperfect control.

In our study, the concept “*cognitive primitives*” refers to observable functional units that represent specific movement patterns and strategies in the context of the maze task, rather than classic motor primitives that are often defined in terms of kinematic patterns or quantitative descriptions that capture characteristic features of human movements^56^. This conceptualization allows us to link the observed behavior to the practical strategies employed by participants to solve the task. The kinematic data, including 3D positions and velocities of the marble and maze tilt angles, were used to segment these primitives, providing a symbolic representation of each trial motion and allowing us to analyze the sequence and frequency of different strategies used.

The original set of primitives was successfully tested and used in implementing actuator movements in a robot humanoid within a learning framework to complete a similar maze task^46,47,57^. Bentivegna's framework, inspired by learning and deriving sub-goals to complete a task from observing human performance^58^, has been used in further research to enable robots to learn and perform complex movements^59–62^.

To validate these cognitive primitives, we ensured that the segmentation process was consistent across different trials and participants. In our tests, our algorithm succesfully identified and categorized each of the proposed primitives, mapping the participants' movements to the corresponding primitive. We applied a systematic method for extracting these primitives from the maze navigation data, using the kinematic data of the sphere and the maze to define the boundaries and characteristics of each primitive. This validation process included testing for reliability and consistency, ensuring that the identified primitives accurately represented the participants’ strategies and movements. However, since the enhanced set of primitives is being tested for the first time in this experiment, we acknowledge that the complete set of primitives requires further testing and validation to be considered final.

The cognitive primitives in the maze task used in our study are defined as follows:

- *Guide* The ball is rolled from one location to another without touching any walls.
- *Roll to Corner* The ball rolls along a wall and stops when it hits another wall.
- *Roll from Wall* The ball rolls along a wall and then maneuvers away from it.
- *In Corner* The ball is moved into a corner, and the board is then almost leveled to allow the next primitive to move the ball out of the corner.
- *Random Bounce* The ball bounces around randomly with little or no apparent control.
- *Roll along Wall* The ball rolls along the wall continuously.
- *Controlled Bounce* The ball bounces off a wall once before exiting a particular section of the maze.
- *Roll to Wall* The ball rolls to a wall and then rolls along the wall.
- *Steady* A variant to the Guide primitive, the ball rolls forward without touching any walls and then returns to a similar position.
- *At Rest* The ball is motionless for a defined period of time.

We use the combined set of primitives as the foundation for obtaining a task performance metric to inspect the performance development of the participants throughout the three days of practice.

### Maze task performance

We employed the cognitive primitives to evaluate the performance of the maze task over a 3-day training period, utilizing our developed primitives and corresponding software to quantify performance precisely. This approach ensured a detailed and accurate measurement across participants, offering comprehensive insights into skill acquisition and task efficiency. A linear regression model was applied to the pooled data to elucidate these aspects further.

Substantial differences were observed between subjects in their initial and final performance and intertrial variability. The regression lines were plotted for visual representation, including their 95% confidence intervals, addressing any potential outlier biases.

The trend analysis revealed consistent improvement in maze task performance over the training period. This enhancement was substantiated by an increase in the performance score to complete the maze, thus reflecting an increase in efficiency and proficiency as the training progressed. Specifically, the linear regression model yielded a significant positive slope, revealing that the score increased as the number of days increased.

The applied model was defined as $y \sim 1 + x1$, with estimated coefficients for the intercept at 0.57071 (SE = 0.0059281, tStat = 96.272, *p* value < 0.001), and for $x1$ at 0.0001016 (SE =  5.3374e−06, tStat = 19.035, *p* value < 0.001). The model was fitted to 1923 observations, with a root mean squared error (RMSE) of 0.13 and an R-squared value of 0.159 (adjusted R-squared of 0.158), indicating that approximately 15.9% of the variance in the dependent variable $y$ was explained by the independent variable $x1$.

The F-statistic for the model against a constant model was 362, with a highly significant *p* value (3.83e−74), affirming that our specific model provided a significantly better fit to the data than an intercept-only model.

**Figure 3:**
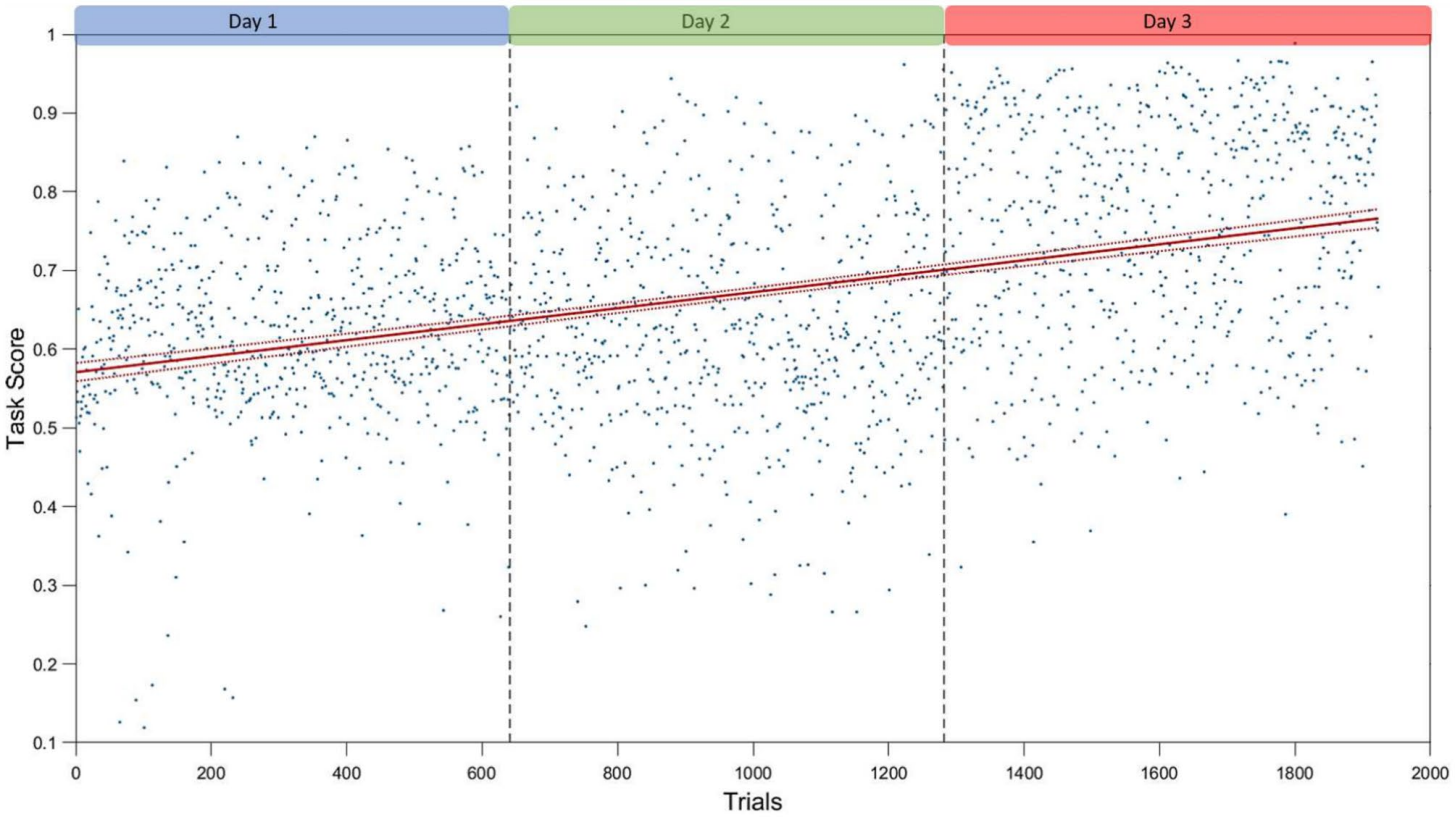
Linear regression analysis of the maze task performance over a 3-day training period. The plot shows the total performance scores of all participants across the training days, with the linear regression line overlayed. The regression line, along with its 95% confidence intervals, illustrates the consistent improvement in performance, reflecting enhanced efficiency and proficiency in the task as the training period progressed.

These statistical outcomes, shown in Figure 3, demonstrate a robust correlation between the cognitive primitives and the observed performance, validating the efficacy of our primitives and software in measuring the maze task performance. The total performance scores of all participants during the 3 days, along with the linear regression overlaid on them, further substantiate this relationship.

**Figure 4:**
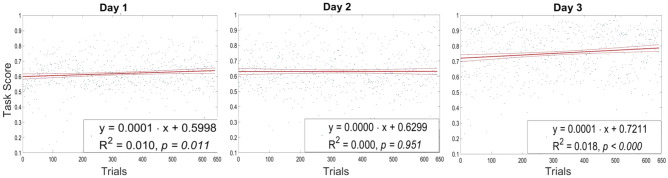
Linear regression analysis of maze task performance across a 3-day training period. The plots show the task performance scores of all participants for each day, with the linear regression line and its 95% confidence intervals overlayed. Day 1 (left) shows a slow improvement in the task $R^2 = 0.010, p = 0.011$. Day 2 (center) indicates no significant change with $R^2 = 0.000, p = 0.951$. Day 3 (right) shows a more pronounced improvement with $R^2 = 0.018, p < 0.000$. These results illustrate the changes in task performance.

Further analysis of the performance scores across the 3 days provided additional insights. The linear regression analysis, as shown in Fig. 4, depicted the task performance scores of all participants for each day, with the regression lines and their 95% confidence intervals overlaid. Specifically, Day 1 (left) showed a slight improvement with $R^2 = 0.010, p = 0.011$. Day 2 (center) indicated no significant change with $R^2 = 0.000, p = 0.951$. Day 3 (right) showed a more pronounced improvement with $R^2 = 0.018, p < 0.000$. These results illustrate the changes in task performance, reflecting enhanced efficiency and proficiency in the task over the training period.

### Joints movement variability

In our study, we aimed to understand the dynamics of key joints engaged in the maze task: maneuvering a sphere from the left to the right side of a maze. Recognizing the potential influence of this specific task on joint activity, we analyzed the 1-s joint velocity profiles surrounding the peak velocity. We focused on the evolution from the initial learning phase on day 1 to a more consolidated stage on day 3, providing insights into how joint coordination changes with task proficiency.

Our analysis revealed that the left wrist, followed by the right wrist, showed larger velocity profiles compared to other joints, indicating their dominant roles in task performance. This is likely because the wrists, being closest to the maze, are crucial for the intricate maneuvering the task requires. Interestingly, as the participants became more skilled at the task, we observed a general increase in velocities for all joints from day 1 to day 3, except for the wrists. Rather than increasing their speed, the wrist joints showed a refinement in movement, suggesting a more efficient strategy was adopted with practice. Moreover, our findings unveiled that the largest variance on the third day, relative to the first day, was observed in the left wrist. Interestingly, this high variance was mirrored in the right shoulder, left hip, and both elbow joints, suggesting these joints too play a substantial role in the learning process and overall task execution.

**Figure 5:**
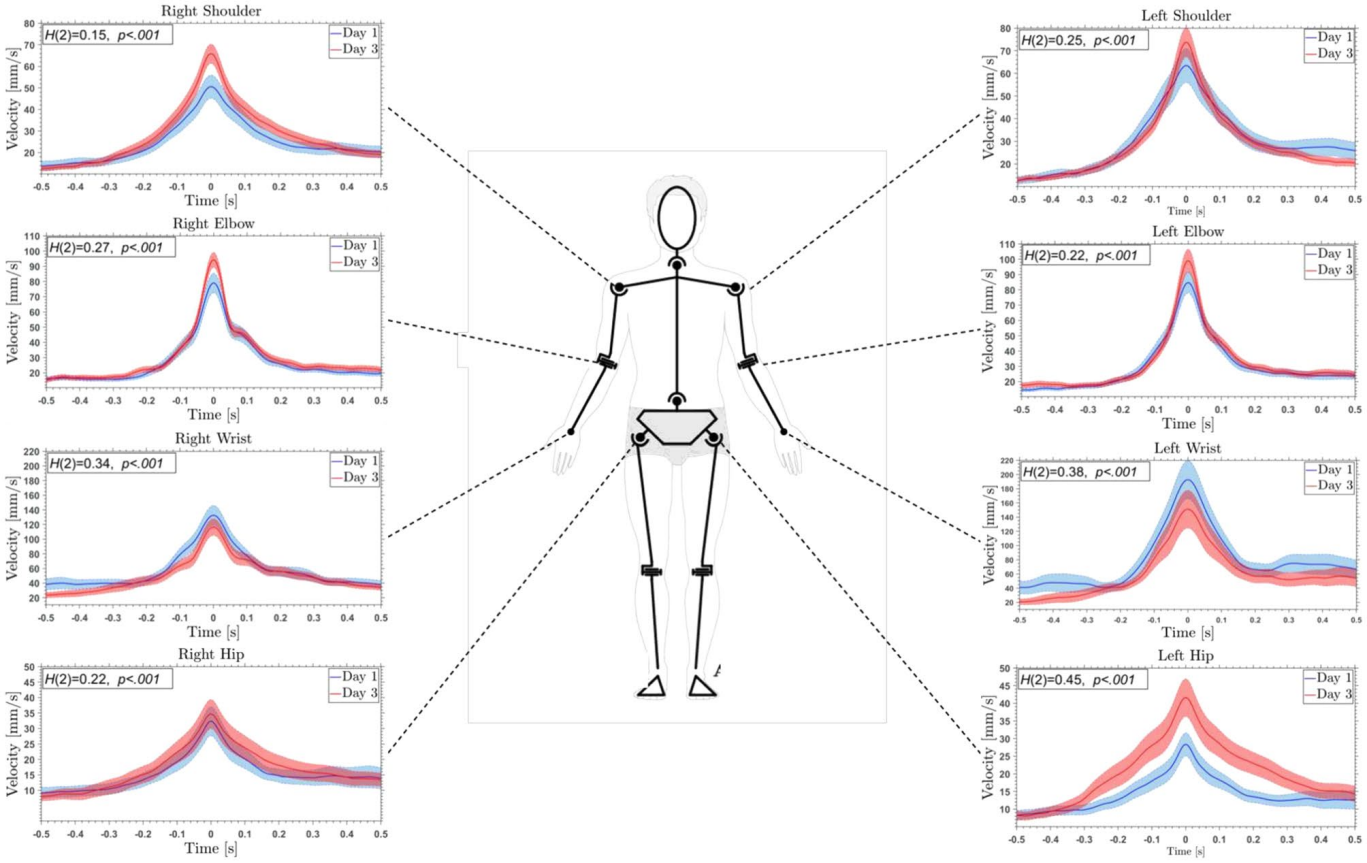
Joint velocity profiles of the upper-body. The 1-second velocity profiles of eight specific joints: Right Shoulder, Right Elbow, Right Wrist, Right Hip, Left Shoulder, Left Elbow, Left Wrist, and Left Hip, centered around the peak velocity. Day 1 is depicted by the blue curve, while Day 3 is illustrated by the red curve. Each curve encompasses a temporal window extending 0.5s before and 0.5s after the maximum peak velocity of every trial, representing averaged measurements taken across multiple subjects and during each day. The accompanying shaded areas around each curve signify the mean and the standard error. For clarity and orientation, each graph is linked to its respective joint on a centrally placed human figure.

Furthermore, significant differences were observed in joint velocities between day 1 and day 3 using the Kolmogorov–Smirnov Test. This highlights the clear evolution in joint coordination and task strategy with continued practice. However, given the exploratory context of our study, we present all statistical tests with caution and chose to display the results graphically in Fig.  5 for clarity, rather than in the main text.

It’s important to note the robustness inherent in our experimental design. All participants performed the task standing in front of the same table and using the same maze. This consistent setup effectively minimized the potential influence of the participants’ body size on the joint velocity profiles, bolstering the reliability of our findings. In our study, we prioritized precision and accuracy as the primary constraints. In terms of precision, we instructed participants to minimize the number of times the ball contacts the walls of the maze. Additionally, the ratio of contact time to total time was to be minimized. This approach aims to ensure that rolling the ball along a wall, which counts as a single contact, incurs a greater penalty than a controlled bounce off a wall. To assess the accuracy of mastering the maze, we counted the number of trials the participants were able to bring the sphere to the final goal position without falling into a pit. We considered every trial executed until the end as an accurate trial; every other trial that had to be interrupted due to falling into a pit was deemed inaccurate. Based on the collected data, the mean group accuracy for each day was calculated.

### Coordination between joint pairs

In examining the coordination between joint pairs and the temporal evolution of motor coordination, we conducted a pairwise cross-correlation analysis of six key upper-body joints: the left shoulder, right shoulder, left elbow, right elbow, left wrist, and right wrist. This analysis was performed for Day 1, representing the initial stage of motor learning, and Day 3, symbolizing a more consolidated phase. The Spearman rank correlation coefficients were calculated to evaluate the correlation between the movements of each joint pair.

For day 1, the correlation coefficients spanned from 0.38 to 0.75. The joint pairs of the right elbow and right wrist, along with the left elbow and left wrist, showcased the highest correlation, with coefficients of 0.75 and 0.74, respectively, indicating strong synchrony in their movements. Notably, the pairs comprising the left shoulder and right wrist demonstrated the weakest correlation (0.38), followed by the right shoulder and left wrist (0.41). Interestingly, pairs comprising the left shoulder and left wrist (0.43), and the right shoulder and right wrist (0.45) also showed comparatively lower correlation levels.

**Figure 6:**
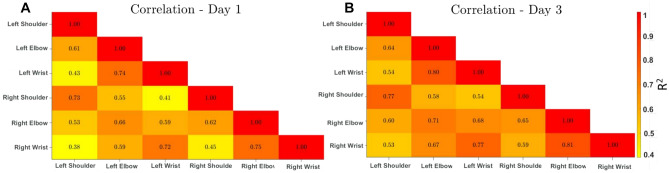
Correlation matrices showcasing the relationships between various body parts (Left Shoulder, Left Elbow, Left Wrist, Right Shoulder, Right Elbow, Right Wrist). (**A**) represents Day 1 and (**B**) represents Day 3. The color spectrum, ranging from red (higher correlation) to yellow (lower correlation), illustrates the intensity of relationships. Notable shifts in correlations from (**A**) to (**B**) can be observed, such as the strengthened relationship between the Left Elbow and Left Wrist, and between the Right Elbow and Right Wrist.

On day 3, an overall increase in the correlation coefficients was observed across all joint pairs, with values ranging from 0.53 to 0.81. This suggests enhanced joint coordination as motor learning progressed. The joint pairs of the right elbow and right wrist, as well as the left elbow and left wrist, retained the highest correlation (0.81 and 0.80, respectively). Regardless of the side, the joint pairs involving the shoulder and wrist increased their correlation to the same level (0.77), indicating consistent coordination between these joints throughout the learning period. Meanwhile, all pairs involving a shoulder and an elbow, irrespective of the side, revealed an increased correlation, indicative of improved coordination, albeit relatively low compared to the other joints. The detailed correlation coefficients for both days and their comparison are visually presented in Fig. 6.

### Thumb pressure distribution

We conducted a comparative analysis between the average pressure images of the right and left thumbs recorded on Day 1, Day 2, and Day 3. We computed the centroids representing high-pressure regions for each day’s tactile images. To enhance our visualization of pressure distribution, we overlaid an ellipsoid, approximating the shape of these regions, on the averaged tactile images.

**Figure 7:**
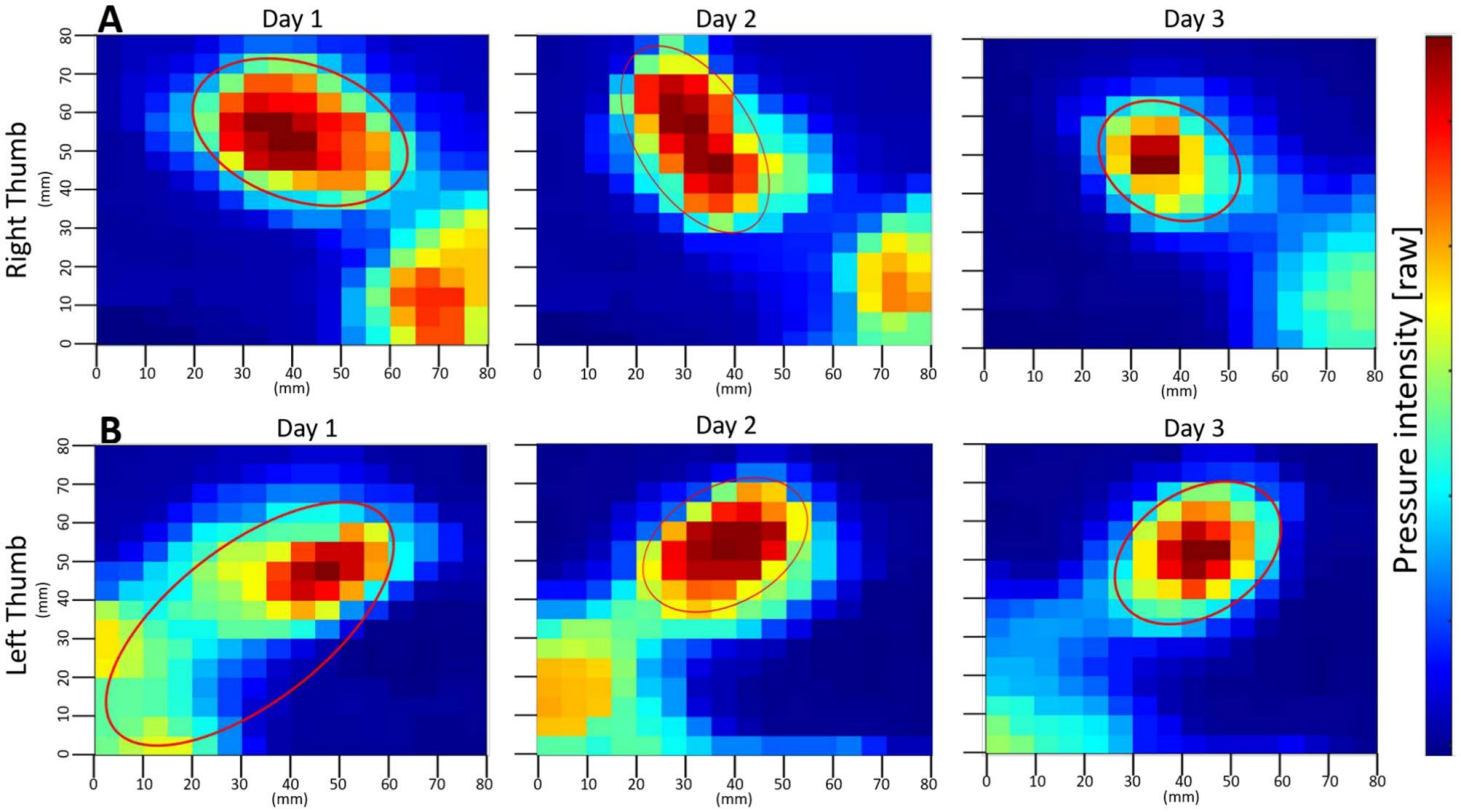
Comparative pressure intensity maps of the right (**A**) and left (**B**) thumbs on Day 1, Day 2, and Day 3. Centroids highlighted by ellipsoids capture the primary regions of pressure concentration. Both thumbs show a modification in the intensity and distribution of pressure from Day 1 to Day 2, and then to Day 3, with the right thumb’s pressure becoming more centralized and the left thumb’s peripheral pressure diminishing.

On the first day, the right thumb demonstrated two pronounced regions of high pressure. The primary region, which exhibited the most substantial pressure, was located near the center of the thumb’s surface. As a result, we chose this centrally positioned centroid for the ellipsoid overlay, effectively representing the major pressure distribution. By the third day, pressure application had shifted markedly, with the peripheral pressure in the corner region significantly reduced and a concentration of pressure at the thumb’s center. This shift, seen in Fig. 7A, suggests a reorientation of the high-pressure zones. Furthermore, a comparison of the ellipsoid areas across the 2 days revealed a significant decrease in size. As substantiated by a one-way ANOVA, there was a significant difference in the mean area of tactile responses between day 1 (M = 60) and day 3 (M = 55.8), F_(1,862)_ = 4.1608, *p* < 0.01.

An analogous trend observed in the right thumb was noted in the left thumb. On the first day, the centroid was somewhat centrally positioned; however, the region of pressure extended towards the lower-left corner of the image, forming a contiguous area. As a result, the overlaid ellipsoid encapsulated this entire region. By the third day, the pressure had become predominantly centralized, with the peripheral corner region no longer contributing to the pressure distribution. This shift in pressure application towards the thumb’s center, is depicted in Fig. 7B. A one-way ANOVA confirmed a significant change in the mean area of tactile responses between day 1 (M = 62.9) and day 3 (M = 56.6), F_(1,912)_ = 7.1921, *p* < 0.01.

## Discussion

In this study, we introduce a novel paradigm for studying the dynamics of motor learning and task performance, specifically within a maze game context. We designed a unique platform tailored for this purpose where we combine the dynamic intricacies of navigating a sphere through a maze with the added complexity of a bimanual task. This approach seamlessly merges the dynamic yet constrained nature of the maze game with real-time motion capture of the human upper body, complemented by state-of-the-art tactile sensors^38^. Our results provide new insights into naturalistic bimanual motor learning, highlighting task performance, upper-body joint movement variability, and the evolution of thumb pressure distribution dynamics.

The introduction of precision and accuracy as constraints within the maze task effectively steered participants’ learning. Participants were instructed to reduce wall contacts to a minimum, focusing on limiting the duration of any such contact. This strategy encouraged continuous refinement of their navigation techniques, fostering a blend of rapid movement and careful strategy. Thus, these constraints proved to be efficient in enhancing skill development and engagement with complex motor tasks^63^.

Our experimental framework allowed participants to execute self-paced, unconstrained movements in the maze without artificial cues such as go signals or time-based scoring. This approach ensured they received authentic somatosensory feedback, thereby minimizing external influences that could potentially skew their learning trajectory. Contrasting such real-world complex bimanual tasks with their simplified virtual counterparts has shown markedly different learning trajectories^64^. These differences emphasize the need for caution when extrapolating results from simplified virtual tasks, considering the high-dimensional, nonlinear, and hierarchical nature of human behavior. Thankfully, advancements in data recording and analysis technologies now enable more in-depth explorations of real-world behavior, although challenges remain.

### Maze task performance and the role of primitives

In order to understand the maze task performance, we were required to delve into movement primitives. Movement primitives are foundational motor patterns that serve as the building blocks for complex motor behaviors^54,55^. Drawing from the original primitives framework by Bentivegna et al.^46,47,65^, we expanded the set of movement primitives to capture a broader spectrum of task movements in our bimanual maze maneuvering, covering both proficient maneuvers and mistakes. Our additions, including “Random Bounce”, “Controlled Bounce”, “Roll Along Wall”, “Roll to Wall”, “At Rest”, and “Steady”, are now referred to as ‘cognitive primitives’, thereby covering the entire set of actions in task execution. These cognitive primitives offered a unique lens through which we could observe the evolution of participants’ strategies. For instance, over the days, there was a marked decline in the use of the “Random Bounce” or “In Corner” primitive and an increase in the “Steady” and “Guide” movements, suggesting that participants were gaining better control over the sphere and making more deliberate choices. This manifested in smoother movements and trajectories, successful maze completions, and reduced errors.

This transition to cognitive primitives originates from the understanding that the original maze primitives were specifically tailored for the maze task and did not consider how they might represent broader cognitive constructs or processes in the brain. We propose that the concept of cognitive primitives can be adapted to elucidate phenomena in various sensorimotor tasks. Nonetheless, further research is essential to expand on this concept and investigate how these representation primitives might be applicable and meaningful in different contexts.

Our trend analysis revealed a consistent improvement in maze performance over the 3-day span. There was a marked correlation between our enhanced primitives and task performance, suggesting that these primitives can be used as tools for in-depth motor learning analysis. As participants fine-tuned their use of these primitives, their overall maze performance improved.

The maze task performance score is directly linked to the cognitive primitives used during the maze task. As participants improved, the frequency of efficient primitives such as “Guide” and “Steady” increased, while the occurrence of penalized primitives like “Roll To Corner” and “Random Bounce” decreased. This relationship is reflected in the performance scores, where fewer errors lead to higher scores. For example, achieving a score close to 1.0 typically involved minimal wall contact, primarily comprising “Guide” primitives. Conversely, lower scores were associated with frequent wall contact and more penalized primitives.

Nevertheless, despite the observable improvement in performance over the course of the study, we also noted high levels of variability, both inter-individual within sessions and between participants across the experiment. This is not merely a consequence of motor learning but an intrinsic aspect, especially in tasks with a high degree of freedom^66–70^. Maselli and colleagues argue in their research on naturalistic throwing that such significant inter-individual differences in behavior, rather than being averaged out, offer valuable insights into learning and adaptation^71,72^. A similar perspective arises from research on naturalistic catching, where individualized strategies led to varying levels of performance, thereby highlighting the existence of multiple ‘correct’ ways to accomplish the task^73,74^.

Moreover, the high dimensionality of our maze task offers an intricate landscape of multiple possible solution combinations for task completion. This complexity, even after participants attained a degree of proficiency, notably by the third day of practice, seemed to perpetuate an explorative phase, pushing them to continually probe and experiment with alternative motor strategies^75^. Such findings are supported by ideas in reinforcement learning^76,77^, which suggest that baseline variability increases exploration, which in turn facilitates learning. Interestingly, motor variability has been demonstrated to facilitate learning in supervised error-based learning tasks as well^78^, indicating a broader role for variability in the context of motor learning. However, this ongoing exploration might have a double-edged nature. On the one hand, the variability in motor performance serves as a vital feedback mechanism, enabling individuals to adapt, refine, and improve their movements by examining various patterns and their outcomes^79–81^. On the other hand, this persistent exploration may also lead to sporadic dips in performance at more advanced stages, as participants might embrace riskier, suboptimal strategies in their quest for proficiency^51,82^.

### Body dynamics

Our analysis of the joint kinematics during the task revealed significant adaptations in motor control strategies as participants transitioned from novice to proficient stages. Unexpectedly, we observed that from Day 1 to Day 3, instead of adhering to the classic speed-accuracy trade-offs (see Heitz for a review^83^), increased velocities in proximal joints like the shoulders were consistent with improved task proficiency^84,85^. This proficiency did manifest distinctively in the wrists—the joints closest to the maze—where instead of an increase in velocity, more refined, lowered velocities movements emerged, indicating a shift toward efficiency with repeated practice^86,87^.

These findings diverge from traditional models of motor task learning^88–90^, which suggest that motor performance can be optimized by reducing or mitigating the inherent noise in the CNS. Our results suggest that during skill acquisition, the central nervous system (CNS) employs a nuanced approach to optimizing motor control. Instead of employing a specific movement pattern for all joints or exerting rigid control over each joint’s movement, the CNS orchestrates movements such that if an action from one joint results in an undesired outcome, other joint movements compensate for minimizing the error^91,92^. As participants progress in task proficiency, different joints likely assume distinct roles in response to the movement’s demands. For instance, proximal joints, like the shoulders, might be responsible for rapid gross movement positioning, whereas the wrists, with their more refined movements, might address the precision demands of the task^93,94^. Moreover, bimanual tasks, due to their inherent complexity, could introduce unique dynamics compared to traditional unimanual tasks^95,96^. Such tasks, necessitating the synchronization of both limbs, might prompt the CNS to employ a hand coordination control strategy, wherein the observed velocity differences between the wrists and other joints are systematically orchestrated for optimal task execution^85,97^.

Furthermore, the differing movement behaviors between proximal and distal joints may indicate the relevance of various feedback mechanisms in motor learning. While rapid movements might stem from proprioceptive feedback from larger muscle groups, tactile and visual feedback might heavily influence the wrists’ refined movements, ensuring a more deliberate motion^98–100^.

From a neuroscientific perspective, the brain’s inherent plasticity is accentuated when faced with the demands of mastering complex motor tasks (see^101^ for a review). As participants mastered the task, the central nervous system might have undergone rapid neural reorganization and prioritized efficiency over raw speed in regions necessitating precision, like the wrists. This adaptive behavior ensures that while the broader movement is executed promptly, the finer details of the task are receiving enough attention, optimizing the balance between speed and accuracy^102^.

Concurrently, our analysis revealed significant adaptive changes in joint behaviors, such as those observed in the left hip and right shoulder, over the course of learning. These joints seemed to undergo functional recalibrations, optimizing their role in the coordination necessary for the task. These adaptations were captured statistically through the Kolmogorov–Smirnov test, highlighting an evolution in joint coordination that emerged with practice. While we interpret these statistical tests cautiously due to the exploratory nature of the study, they still elucidate the behavioral dynamics observed on the joints across days under the skill acquisition process^103,104^.

Moreover, the pairwise cross-correlation analysis across key upper body joints-encompassing shoulders, elbows, and wrists-unveiled complex coordination patterns that evolved during the training period. While initial synchrony (as evidenced on Day 1) within right and left elbow–wrist pairs was strong, by Day 3, we noticed a pronounced improvement in coordination, suggesting an innate adaptability within the motor system and its ability to fine-tune itself through repetitive, iterative practice^41,82,105,106^.

This observed improvement aligns with studies that showed that recruiting additional degrees of freedom, such as pendulum swinging^107,108^, torso twisting or extra arm movements^108,109^, can stabilize challenging bimanual coordination patterns. According to the dynamical systems theory of movement coordination, incorporating these additional degrees of freedom provides greater flexibility to the system, allowing it to explore a larger area of its state space and converge on the intended bimanual pattern^110^. This functional engagement of extra degrees of freedom acts as control parameters stabilizing the coordination pattern. Our findings support this theory, as the observed increase in coordination between the wrists and shoulders over the training period likely reflects the motor system’s ability to recruit and utilize additional degrees of freedom to achieve and stabilize the complex bimanual task^110^.

Despite these insights, we acknowledge the exploratory scope of our study, necessitating a cautious interpretation of the results. Our research into bimanual maze navigation dynamics is still preliminary. Nevertheless, the evidence gathered highlights the central nervous system’s instrumental role in organizing sophisticated inter-joint coordination strategies, essential for a complex bimanual motor task^78,111–113^.

### Thumb pressure

Thumb pressure, an often-overlooked aspect of motor tasks, offers a profound opportunity to learn insights into sensorimotor adaptations^34,114^. Our comparative assessment between Day 1, Day 2, and Day 3 showcased significant thumb pressure adaptations as participants evolved during the task execution. Throughout the task performance, participants exhibited varying degrees of pressure applied by the thumb, which correlated with their overall task performance experienced during the progression of the early skill acquisition stage. This observation resonates with the notion of rapid sensorimotor adaptation, where the sensory feedback from initial task performance feeds into adjustments in subsequent motor commands^41,66^.

On Day 1, the tactile images highlighted two high-pressure regions in the right thumb, located centrally and moving towards the thumb’s pad. On Day 2, the high-pressure region around the pad dissipated, with the pressure concentrating around the center. By Day 3, participants exhibited a pronounced shift in pressure application. The high-pressure zones reoriented, concentrating more at the thumb’s center, reducing significantly at the peripheries. This centralization of force application indicates a strategic recalibration of motor control, possibly driven by the subjects’ enhanced task familiarity and comfortability^34,66^, observed in precision motor tasks^115,116^.

The left thumb mirrored this pattern, with initial pressure application extending towards the periphery, subsequently receding to a more centralized distribution by Day 2, and finally focusing its high pressure centrally on Day 3. This uniformity in adaptation between both thumbs underscores a bilateral refinement in motor control, potentially indicative of a holistic improvement in task execution strategy^117^.

The decrease in pressure area and the repositioning of pressure application away from the peripheries suggest a refined focus on motor precision^63^). This behavior is particularly crucial for our maze task, where precise thumb movements are essential for fine manipulation and successful task completion. In tasks demanding fine manipulation, the role of tactile feedback serves as a crucial informant for iterative fine-tuning of motor outputs^34,100,118^. The shifts in thumb pressure distribution, suggest that as participants become more acquainted with the task, their neuromotor system undergoes strategic recalibrations leading to refined motor strategies^41,66^.

These observed changes in thumb pressure are directly relevant to our primary hypothesis regarding motor skill acquisition and adaptation in bimanual tasks. Specifically, the reduction and centralization of thumb pressure over the course of the experiment reflect the participants’ increasing proficiency in utilizing fine motor control and sensory feedback to optimize their performance. This adaptation is hypothesized to involve neural plasticity, where repeated practice and sensory input lead to the strengthening of neural pathways associated with precise motor control^41,119^.

Moreover, the modifications in thumb pressure distribution coincide with the corresponding changes in kinematic patterns across the body, as observed in joint coordination and movement strategies, highlighting the central nervous system’s capacity for integrated, multisensory optimization^120–124^. As participants practice, the neural circuits involved in motor planning and execution likely become more efficient, leading to more coordinated and stable bimanual movements. This integrative approach to motor adaptation underscores the importance of sensory feedback in driving the fine-tuning of motor outputs, ultimately resulting in enhanced motor skill proficiency. Thus, we interpret the evolution of thumb pressure distribution depicted in our results not only as a marker of mechanical adjustment but also as a reflection of the underlying neural and motor adaptations.

While our findings are insightful, they open avenues for further inquiry. Subsequent studies could delve deeper into the longitudinal aspects of this pressure adaptation, exploring beyond the early stages of learning to understand how these strategies evolve with continued practice^125^. Emphasizing the value of tactile feedback and the integration of tactile information in these processes could not only have implications for skill acquisition but also enhance our understanding of sensorimotor control dynamics^2^. Such insights have potential implications for rehabilitative practices and ergonomic designs and for improving execution efficiency in various applied settings.

## Methods

### Participants

Twelve participants from a local university (5 female, 7 male; ${M^{age}}$ = 21.38 years, *SD* = 1.92), with little to no prior experience in similar maze games, participated in the present study. Participation was voluntary, and all participants provided written and informed consent prior to their participation, as well as for the use of any images or videos depicting them included in an online open-access format. They self-reported being healthy, having normal or corrected-to-normal visual acuity, and having no known cognitive or neurological problems. Additionally, all participants self-reported being right-handed, although no handedness questionnaire was applied. The experiment was conducted in accordance with the ethical standards established in the 1964 Declaration of Helsinki and its 2013 revision, and was approved by the Ethics Review Board of Bielefeld University.

### Apparatus and maze design

The experiment took place in the Manual Intelligence Laboratory (MILAB)^37^, which features 14 Vicon MX3+ cameras (Vicon Motion Systems Ltd., Oxford, UK (VICON Nexus 2.1, https://www.vicon.com/software/nexus/)) operating at 200 Hz for motion capture. The maze incorporated tactile sensors to measure the pressure exerted by participants during task performance^38^. The maze measures 17 cm $\times$ 15 cm, with tactile sensor pads on the left and right, each sized 9 cm $\times$ 9 cm (sensor area 8 cm $\times$ 15 cm, including a 5 mm frame). The overall weight of the maze, including the tactile pads, is 650 g. A contact microphone was placed beneath the maze to detect contact collision events, and a high-speed Basler camera operating at 200 Hz was used for data recording. The participants had reflective markers attached directly to their bodies using double-sided tape, following the Vicon Nexus Plug-in-Gait model marker set and modeling as described in previous studies^126^ and using standardized palpation methods on anatomical landmarks^127^. For detailed visualization of the marker placement see Fig. 1B. This approach ensured similar marker adherence and accuracy compared to Velcro suits^128^.

The maze was designed with an array of features to challenge participants, including straight sections, turns, cross-junctions, and terminal sections. Pits were strategically positioned along both potential paths (upper and lower) to add difficulty (see Fig.  1C for a detailed view).

### Experimental setup

Participants stood in front of a table (height = 880 mm, depth = 760 mm, width = 1060 mm), holding the maze with both hands while performing the task. They underwent a 10-min familiarization phase with the maze before the main experiment. The goal was to guide a sphere from the starting point to the target position, avoiding pits. Participants could perform as many trials as desired during this phase. A single trial was considered completed when the sphere reached the target position or terminated if it fell into a pit, requiring a restart from the starting point. To ensure equal exposure to both paths through the maze, participants’ time on the upper and lower paths was balanced. They were informed that there was no time constraint for completing each trial. Following the familiarization phase, the main experiment began. For three consecutive days, they engaged in a 5-min warm-up and a 20-min learning session, adhering to the guidelines of avoiding any wall contact and reaching the target position. Participants spent 10 min on one path before switching to the other for another 10 min, with no time constraints for individual trials. No feedback was provided except for the visible consequence of trial termination upon falling into a pit.

During the learning process, we recorded subjects’ upper-body movements using the Vicon Nexus Plug-in-Gait model, tracked the sphere on the maze, and collected tactile pressure data for each trial for all participants. This comprehensive data collection allowed us to analyze the participants’ motor learning, movement efficiency, and pressure exerted on the maze as they navigated the sphere toward the target position while avoiding wall contact and the pits. Each participant completed approximately 175 trials during the experiment over 3 days.

### Maze sphere tracking

We tracked the movement of the sphere in the maze using a high-speed Basler 602 fc 200 Hz top-mounted video camera (Basler AG, Germany). Our custom-developed software^37^ captured and synchronized the video, high-performance event timer, and camera frames. The software converted the images from the camera’s proprietary CVB format to an uncompressed Audio Video Interleave (AVI) format for further processing. Video frames were stored as AVI files to preserve the relationship between pixel changes and timings, while the computer’s real-time clock time-stamps were recorded in a text file. During subject-paced trials, the experimenter observed the participant and pressed the spacebar key as an additional trigger event for the time-stamps text file, later used for segmenting the continuous data stream into trials.

To facilitate tracking, the metal sphere was painted bright red, and four Vicon markers were placed on the maze’s corners. The Basler camera, calibrated with the Vicon setup, worked in conjunction with custom C++ software using the Image Component Library (ICL)^129,130^ (version 10.0.2, available at https://iclcv.github.io/) to calculate the sphere’s position offline from video recordings. This system was further enhanced by overlaying a model of the maze, divided into 21 sections, onto the maze image for precise tracking of the sphere’s movement in each segment (see Fig. 1D). Data segmentation and trajectory extraction of the sphere for each trial were conducted using MATLAB software (R2021a, The MathWorks, Inc., MA, USA, https://www.mathworks.com/products/matlab.html), with a $10\times 10$ pixels bounding box (approximately $20\times 20$ mm) set around the 15 mm diameter sphere to detect its movement onset, acceleration, and deceleration. This comprehensive setup, integrating video recordings, microphones, and maze markers, enabled the offline computation of the maze and sphere’s positions.

### Task performance

In order to compute a task performance score for the maze game, we developed an automated method to segment each trial into sequences of cognitive primitives. We chose to adopt a similar approach as Bentivegna and colleagues^47^ by assigning penalties to specific primitives, rather than utilizing machine learning algorithms^131^ or a heuristic approach^132^. Each trial was segmented into a sequence of cognitive primitives, and each primitive was assigned a penalty based on whether it involved contact with the wall. For example, a “Controlled Bounce” had a penalty score of 0.03, a “Random Bounce” a penalty of 0.06, a “Roll Along Wall” a penalty of 0.12, and a “Roll to Wall” a penalty of 0.09. An ideal trial, composed solely of “Guide” primitives and other non-wall-contact primitives, was assigned a score of 1. For each instance of a wall-contacting primitive, a corresponding penalty was subtracted from this ideal score. Thus, a symbolic representation of each trial was produced, and an overall fitness score was computed by measuring the distance of each trial from the ideal score. Longer strings were not directly penalized, meaning that longer periods of time maneuvering the maze did not affect the score; only the primitives were quantified. The automation of this process facilitated a quick adaptation of our set of primitives and enabled subsequent re-analyses when needed. Importantly, our scoring system did not directly penalize the length of the trial sequences

### Upper-body motion tracking

Kinematic data were recorded at 200 Hz using the Vicon Nexus motion-capturing system, employing the standard Plug-in Gait model. The Vicon Nexus system utilizes a biomechanical model and proprietary algorithms to estimate 3D joint kinematics. The Vicon system has been extensively validated and utilized in tracking real-world behavior across numerous experiments^133–135^.

To extract a velocity profile for each body joint, we differentiated the position data with respect to time and applied a fourth order low-pass Butterworth filter to the raw velocity data to reduce noise. The cutoff frequency was automatically determined based on the velocity profile data and the joint being analyzed, as recommended by Yu et al.^136^. Finally, we exported the velocity profile files and analyzed them using custom MATLAB software (R2021a, The MathWorks, Inc., MA, USA, https://www.mathworks.com/products/matlab.html).

### Maximum peak velocity profile analysis

To analyze the extracted velocity profiles of all joints for each trial across the three days of the experiment, we aligned them around the peak velocity during each trial. This peak velocity is a consistent and informative point in the motion’s critical phase, reflecting movement efficiency and simplifying data analysis. This velocity is calculated in the three-dimensional space, obtaining the magnitude of the joint velocity in Euclidean terms for each joint throughout the entire trial^137^.

We focus on a 1-s window surrounding the peak velocity to examine joint movement dynamics comprehensively^29,138^. This approach captures the moment when the peak velocity occurs across all joints within the same trial, providing insights into participants’ body coordination and control. Although the peak velocity event may vary from trial to trial due to the dynamic nature of the task, it occurs simultaneously on all joints within each trial.

The peak velocity represents the maximum effort and speed exerted by the participant, serving as a crucial indicator of motor control and efficiency. Focusing on this critical phase allows us to track participants’ motor learning progress over the 3 days, identifying significant changes in the velocity profiles. Furthermore, we generated an average velocity profile curve for each day, providing a clearer visualization of the overall trends and changes in participants’ performance throughout the experiment.

### Tactile pressure

With the tactile sensors, we collected pressure values for the thumb finger of both the left and right hands. The sensor’s pressure range was calibrated between 3 and 100 kPa, and the response values ranged from 0 to 4095. Due to the sensor’s characteristics, the response values could not be directly interpreted as a linear measure of the net normal force applied to the sensor. Therefore, we extracted a $16\times 16$ tactile image from the values. This tactile image provided a comprehensive representation of the pressure distribution, which allowed us to analyze the data more accurately. We then preprocessed the tactile image with a median filter to remove any unwanted noise and artifacts^139^. Subsequently, we averaged the tactile values for each trial using custom MATLAB software (R2021a, The MathWorks, Inc., MA, USA, https://www.mathworks.com/products/matlab.html) to create a mean tactile pressure image and produce a heatmap. We then calculated the image centroids, which indicate the center of pressure, using the moments’ method originally described by Hu^140^ and later adapted by Cannata et al.^141^. This method was applied to each tactile image generated, allowing us to approximate the shape of the pressure distribution as an ellipsoid. The computed centroids indicate the location of the high-pressure region, and the ellipsoids were overlaid on the averaged tactile images for each day, providing a clearer visualization of the pressure distribution throughout the experiment.

### Statistical analysis

In our study, we performed various statistical analyses to assess the accuracy of maneuvering the sphere through the maze, evaluate joint movement changes, examine correlations between joints, and analyze differences between the tactile image centroids. We focused on the comparison between day 1 and day 3, as this allows us to capture the most significant changes in skill acquisition, joint coordination, and pressure distribution that occurred throughout the experiment. The significance level for all analyses was set at 5%.

For the 1-s velocity profiles of each joint, we compared day 1 vs day 3 using a Kolmogorov–Smirnov test. This test allowed us to evaluate the statistical difference in the joint velocity profiles between the initial and final performance of the participants.

Next, we obtained the correlation of all the joints for each day, providing insight into the joint coordination patterns throughout the learning process. Correlational analyses were conducted using Spearman correlations.

Finally, we compared the ellipsoids fitted to the pressure distribution in the averaged tactile images for each day. This comparison allowed us to visually assess the changes in the shape and orientation of the pressure distribution throughout the experiment. Additionally, a one-way ANOVA was performed to test for significant differences in the mean area of tactile responses between Day 1 and Day 3. When necessary, the non-parametric Mann-Whitney U test was used to account for unequal sample sizes and non-normal distributions.

## Conclusions

In this study, we introduce a novel maze game platform to simultaneously explore various facets of motor learning within a complex bimanual task. Our expanded set of movement primitives allowed us to observe the shifts in participants’ movement strategies, progressing from common errors to proficient actions. The variability in performance observed among participants highlights the individualized nature of motor learning trajectories in naturalistic experiments. The kinematic analyses, complemented by thumb pressure adaptations, provide a holistic view of the interplay between sensorimotor integration and the central nervous system (CNS). Such an interplay revealed how the CNS adeptly modulates motor outputs in sync with sensory feedback. Moreover, the unveiling of the maze game and its accompanying setup could offer a versatile and innovative tool for advancing future research in motor learning and bimanual coordination. Our results provide new insights into naturalistic bimanual motor learning, highlighting task performance, joint movement variability, coordination between joint pairs, and the evolution of thumb pressure distribution dynamics (Supplementary Videos 1 and 2).

### Supplementary Information

Supplementary Information.

Supplementary Video 1.

Supplementary Video 2.

## Data Availability

Data sets generated during the current study are available from the corresponding author on reasonable request.
