# Supplementary material for: Exploring motor skill acquisition in bimanual coordination: insights from navigating a novel maze task
Source: Sci Rep. 2024 Aug 14;14:18887. doi: 10.1038/s41598-024-69200-1 (PMC11324764; doi:10.1038/s41598-024-69200-1)
Supplement: Supplementary file 1 — Supplementary Information. [file 41598_2024_69200_MOESM1_ESM.docx]

**Additional information: Video demonstrations**

In the supplementary section of this research paper, we have included two video demonstrations, showcasing one complete trial from Day 1 and another from Day 3 for the same participant. These videos provide a detailed representation of the participant’s progression, illustrating the practical aspects of our findings. They capture the evolution of motor skills in a real-world context, highlighting the transition from initial attempts to more refined maneuvering.
